# Helical spin dynamics in Cu_2_OSeO_3_ as measured with small-angle neutron scattering

**DOI:** 10.1063/4.0000305

**Published:** 2025-07-18

**Authors:** Victor Ukleev, Priya R. Baral, Robert Cubitt, Nina-Juliane Steinke, Arnaud Magrez, Oleg I. Utesov

**Affiliations:** 1 Helmholtz-Zentrum Berlin für Materialien und Energie, D-12489 Berlin, Germany; 2 Department of Applied Physics and Quantum-Phase Electronics Center, The University of Tokyo, Bunkyo, Tokyo 113-8656, Japan; 3 Institut Laue–Langevin, 71 avenue des Martyrs, CS 20156, Grenoble, 38042 Cedex 9, France; 4 Institute of Physics, École Polytechnique Fédérale de Lausanne (EPFL), CH-1015 Lausanne, Switzerland; 5 Center for Theoretical Physics of Complex Systems, Institute for Basic Science, Daejeon 34126, Republic of Korea

## Abstract

The insulating chiral magnet Cu_2_OSeO_3_ exhibits a rich array of low-temperature magnetic phenomena, making it a prime candidate for the study of its spin dynamics. Using spin wave small-angle neutron scattering (SWSANS), we systematically investigated the temperature-dependent behavior of the helimagnon excitations in the field-polarized phase of Cu_2_OSeO_3_. Our measurements, spanning 5–55 K, reveal the temperature evolution of spin-wave stiffness and damping constant with unprecedented resolution, facilitated by the insulating nature of Cu_2_OSeO_3_. These findings align with theoretical predictions and resolve discrepancies observed in previous studies, emphasizing the enhanced sensitivity of the SWSANS method. The results provide deeper insights into the fundamental magnetic properties of Cu_2_OSeO_3_, contributing to a broader understanding of chiral magnets.

## I. INTRODUCTION

Recent studies have revealed a multitude of intriguing phenomena related to topological spin textures in cubic chiral magnets such as skyrmions,^1–3^ magnetic hedgehogs,^4,5^ merons,^6,7^ and hopfions.^8^ One of the prominent members of this family of materials is cubic chiral insulator and skyrmion host Cu_2_OSeO_3_, which with its unique spin-wave properties, offers a distinctive platform for applications in spintronic and magnonic devices.^9–11^ Notably, novel phases such as tilted conical spirals and disordered skyrmions have been recently identified in Cu_2_OSeO_3_ at low temperatures.^12–17^ Thus, our focus is on elucidating the temperature-dependent dynamics of helical spin excitations within the field-polarized regime of Cu_2_OSeO_3_. Refinement of its magnetic parameters such as spin-wave stiffness and damping can help fine-tune micromagnetic^18^ and *ab initio*^19^ theories of this remarkable material, which often suffer from a lack of reliable experimental input.^20^

In this study, we explore the spin-wave dynamics in Cu_2_OSeO_3_ using spin-wave small-angle neutron scattering (SWSANS).^21^ High-resolution of SWSANS compared to classical inelastic neutron scattering experiments provide unique insights into low-energy spin dynamics in chiral helimagnets. Furthermore, the insulating nature of Cu_2_OSeO_3_ facilitates the extraction of damping constants, enabling a robust alignment with theoretical predictions. By applying an analytical framework, we offer quantitative assessments of both spin wave stiffness and damping constants across a wide temperature range from 5 to 55 K ( $T_{C}=58$ K^22^) comparing them with previous neutron scattering results.

## II. EXPERIMENTAL

SWSANS measurements were carried out using the D33 setup,^23^ Institut Laue-Langevin (France), using neutrons with a wavelength of λ=5 Å and Δλ/λ=10%. The incoming beam was collimated over the distance of 7.8 m, and the detector was placed at the distance of 6.5 m behind the sample. The acquisition time was 90 min per the SANS pattern. The magnetic field was controlled by the horizontal-field high-temperature superconducting magnet. Two-dimensional SANS data were reduced using the GRASP software.^24^

All SANS data presented in this article were obtained from ∼ 200 mg disk-shaped single crystal containing (001) plane, which has a diameter of 1 cm and thickness of 2 mm. The single crystal was grown using the chemical vapor transport (CVT) reaction as described in Ref. 18. The horizontal magnetic field was always aligned with the [110] crystal axis in the sample plane and perpendicular to the incoming neutron beam. Elastic SANS pattern measured in the helical magnetic phase at zero field in shown in the Appendix [(Fig. 4)].

Measurements were performed for the temperature range 5–55 K spanning through the entire phase diagram of Cu_2_OSeO_3_ including the A-phase^22^ and novel low-temperature phases.^12,13^ At each temperature, SWSANS signal was measured in four magnetic fields above the saturation ( $H_{C2}$) in order to extract the linear dependence of the cutoff angle (see Sec. III for details). Background SANS signal was measured at 2 K and 1 T and subtracted from other data.

## III. THEORETICAL FRAMEWORK

The spectrum of long-wavelength magnons in the fully polarized phase of cubic helimagnets reads^25^

$$\epsilon_{Q}=A_{\text{ex}}{\left(Q-k_{s}\right)}^{2}+g\mu_{B}(H-H_{C2}).$$ (1)

Here, Q is the magnon wavevector, $A_{\text{ex}}$ is the spin-wave stiffness, neglecting the anisotropic interactions, $H_{C2}=A_{\text{ex}}k_{s}^{2}/g\mu_{B}$ is the field of transition between the field-polarized and the conical phase, and $k_{s}$ corresponds to the spiral vector of the conical phase, which is oriented along the field H. Noteworthy, cubic anisotropy and anisotropic exchange significantly influence Cu_2_OSeO_3_ properties.^13,15,26^ However, in the fully polarized phase and our experimental geometry, their main effect is renormalization of $A_{\text{ex}}$ and $H_{C2}$ that preserves the form of Eq. (1). For definiteness, we choose the *x*-axis along H. For Cu_2_OSeO_3_, well below the ordering temperature $T_{C}=58$ K, $H_{C2}∼50$ mT^22^ and $k_{s}=0.0102$ nm^−1^, which corresponds to the spiral pitch *ca.* 62 nm.^27^

The crucial parameter for SWSANS relates the energy of the incident neutrons with the magnetic system energy scale.^21^ Explicitly, we define $\theta_{0}=E_{i}/A_{\text{ex}}k_{i}^{2}$, where $E_{i}=\hbar^{2}k_{i}^{2}/2m_{n}$. Then, for the spectrum (1), the kinematic conditions for the scattering are fulfilled only if the scattering angle in the detector plane $\theta =(\theta_{x},\theta_{y})$ satisfies $\theta_{\text{rel}}^{2}\equiv {\left(\theta_{x}-\theta_{B}\right)}^{2}+\theta_{y}^{2}\leq \theta_{C}^{2}$, where the Bragg angle $\theta_{B}=k_{s}/k_{i}$, and the cutoff angle is given by^21^

$$\theta_{C}^{2}=\theta_{0}^{2}-\frac{g\mu_{B}(H-H_{C2})\theta_{0}}{E_{i}}.$$ (2)

By performing a linear fit to the field dependence of the cutoff angle, crucial parameter $\theta_{0}$ can be readily extracted and, consequently, $A_{ex}$ value can be determined.

Introducing the magnon spectral line broadening Γ allows relaxing the energy conservation law. The physical mechanisms behind this quantity in dielectrics can be magnon–magnon scattering, magnetoelastic coupling, and lattice impurities. In Ref. 28, it was shown that in order to account for the magnon damping, one can use the formula

$$\sigma (\theta )\propto \text{Im}\frac{1}{\sqrt{\theta_{\text{rel}}^{2}-\theta_{C}^{2}-i\gamma }}$$ (3)

for the SWSANS data interpretation. Here, $\gamma =\theta_{0}\Gamma /E_{i}$ is the dimensionless broadening for magnons at the cutoff, with energy $\epsilon =2E_{i}\theta_{0}$ (in practice, Γ also includes a contribution from the device resolution).

## IV. RESULTS AND DISCUSSION

**FIG. 1.**
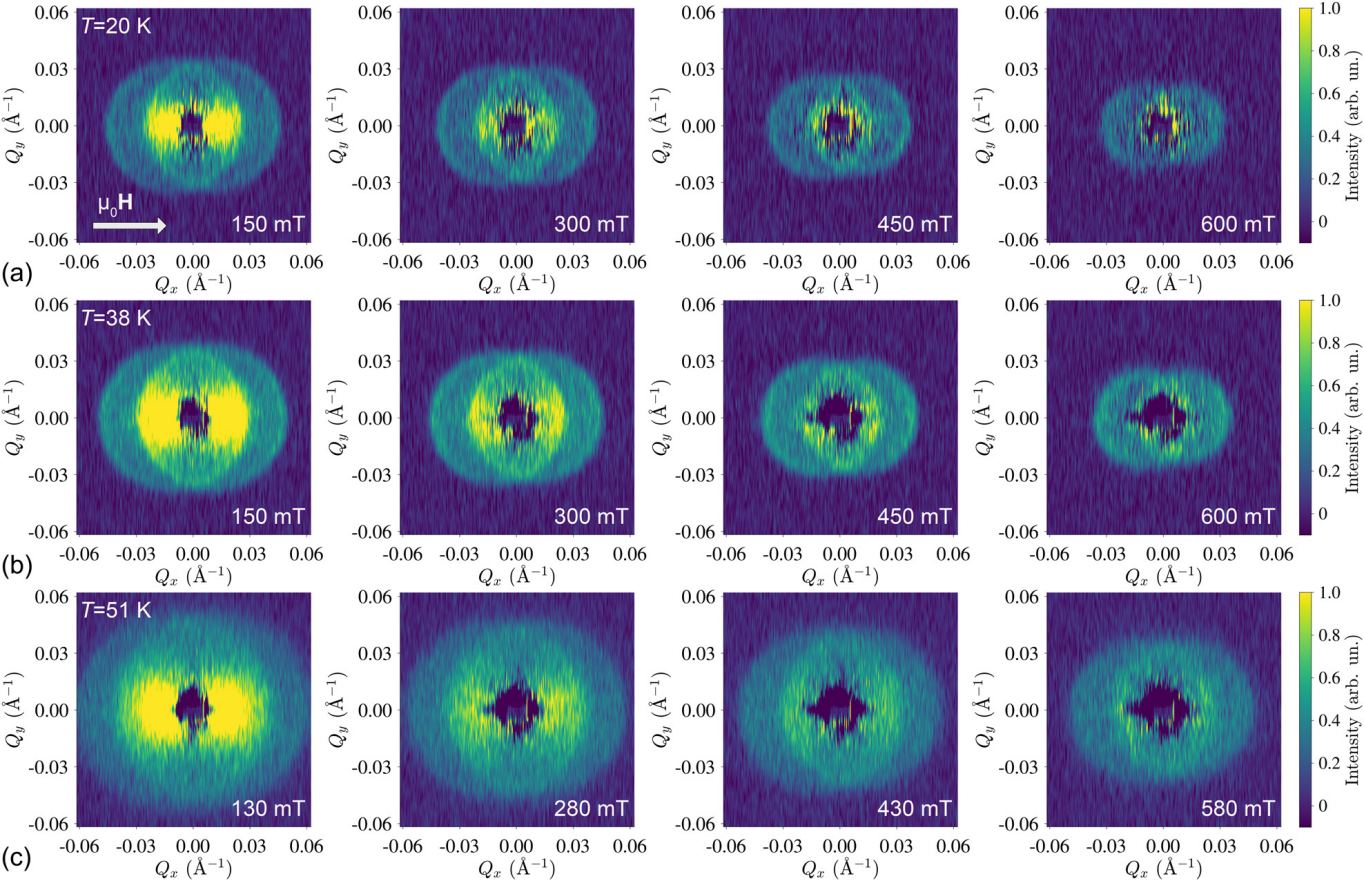
Magnetic field dependencies of SWSANS patterns at (a) 20, (b) 38, and (c) 51 K.

SWSANS patterns from the Cu_2_OSeO_3_ crystal were measured at several temperatures. The intensity of the inelastic scattering is significantly increased compared to the previous study by Grigoriev *et al.*^29^ thanks to the larger sample volume. Typical SWSANS data measured at 20, 38, and 51 K at different magnetic field magnitudes are shown in Fig. 1. Despite the development of a strong exchange anisotropy at low temperatures in Cu_2_OSeO_3_,^26^ its influence is negligible for the present experimental geometry, and its influence on SWSANS will be a subject of our future works. At each temperature, the onset of the shrinking SWSANS circles allows to extract the exchange stiffness parameter $A_{\text{ex}}$. From the two-dimensional SANS images, one can already note that the scattering intensity is somewhat enhanced on the edge of the circle, following the theoretical prediction made in Ref. 28.

**FIG. 2.**
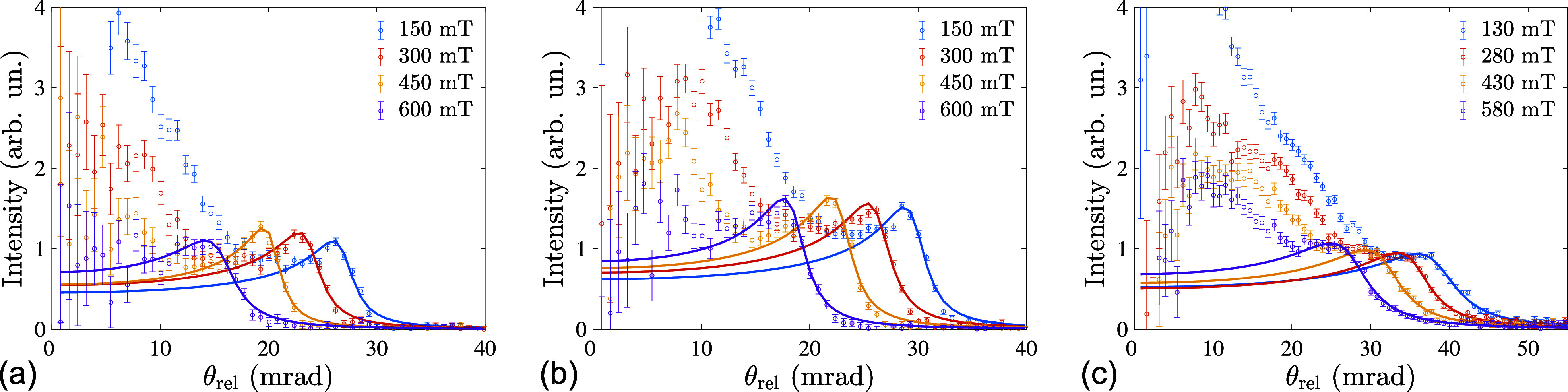
Radially averaged profiles of SWSANS intensity measured at (a) 20, (b) 38, and (c) 51 K and at different magnetic fields. Solid lines correspond to the fit according to Eq. (3).

For quantification of the $A_{\text{ex}}$, SWSANS intensity profiles were extracted for each temperature and field point measured. Typical radially averaged profiles of SWSANS intensity are shown in Fig. 2. Note, that the radial averaging was done from the center of the elastic helical Bragg peaks at the angle $\theta_{B}$. SWSANS radial profiles were fitted to the model according to Eq. (3) (solid lines in Fig. 2). A peak of SWSANS intensity is clearly observed just before the cutoff angle at lower temperatures, which perfectly agrees with the model. The peak has been predicted in Ref. 28; however, it could not be detected experimentally in earlier SWSANS works due to the strong SW damping in metallic chiral magnets MnSi,^21^ Mn_1−*x*_Fe_*x*_Si,^30^ FeGe,^31^ Fe_1−*x*_Co_*x*_Si,^32^ and Co_8_Zn_8_Mn_4_.^28^ In the previous study on Cu_2_OSeO_3_, the bump was not clearly discernible due to the smaller sample size and was also not accounted for by the theory proposed in Ref. 29. However, we would like to point out that it was experimentally observed using polarized neutron scattering on spin waves in amorphous ferromagnets.^33,34^

**FIG. 3.**
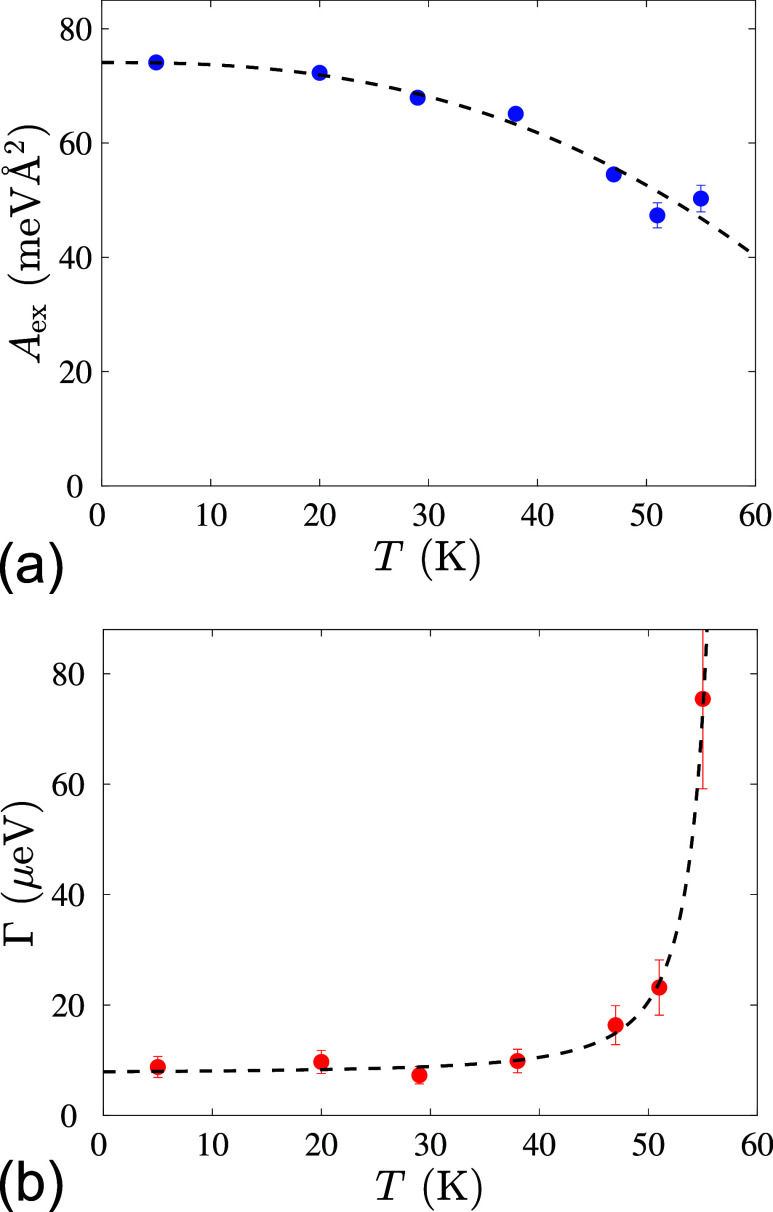
(a) Exchange stiffness and (b) damping parameters of Cu_2_OSeO_3_ obtained from the SWSANS data and modeling. Error bars in the panel (a) are smaller than the symbol size for temperatures below 50 K.

**FIG. 4.**
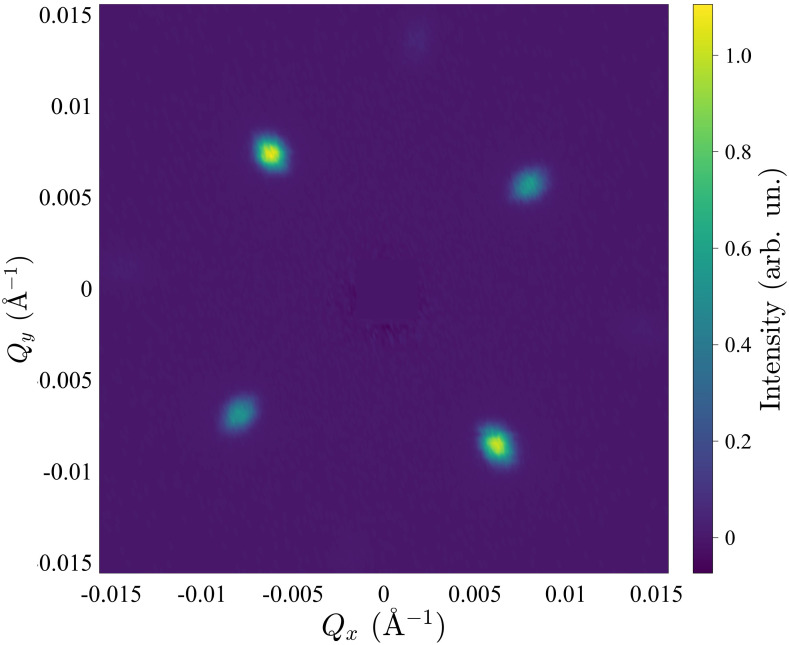
Elastic SANS pattern measured at zero magnetic field at T=2 K in the helical phase.

Exchange stiffness and spin-wave damping parameters obtained from the fit are shown in Figs. 3(a) and 3(b), respectively. The dashed line in Fig. 3(a) represents the fit according to the following law:

$$A=A_{0}\left[1-c{\left(\frac{T}{T_{C}}\right)}^{5/2}\right].$$ (4)

The resultant fit matches the experimental data well. Here, the parameter *c* is a constant of an order of 1, which can be obtained by integrating the magnon interaction and their Bose–Einstein occupation numbers over the entire Brillouin zone, and it cannot be determined without a precise microscopic model. The fitted parameters, $A_{0}=74.1\;\pm \;0.3$ meVÅ^2^ and c=0.42±0.05, align very well with those from the previous SWSANS study reported in Ref. 29. Exchange stiffness parameter in previous cold neutron inelastic scattering experiments was extracted from the parabolic fit of the dispersion curves.^35,36^ Interestingly, a similar stiffness to our value was found in Refs. 35 and 37, while somewhat lower values of 50 meVÅ^2^ were also reported.^36^ We believe that the ultimate resolution of our SWSANS probe resolves the discrepancy in favor of the higher value. Regarding the damping, Fig. 3(b) shows that it naturally increases with temperature, becoming significantly large near $T_{C}$, where magnons cease to be well-defined quasiparticles (in particular, power-law fit reveals $\Gamma \propto {\left(T_{C}-T\right)}^{-2}$ dependence). The broadening of the cutoff angle due to the finite experimental resolution can be estimated as $\gamma_{r}=\theta_{C}\delta \theta$, given the resolution of 4 mrad mainly due to the 10% neutron wavelength bandwidth. Hence, the corresponding minimal measurable damping parameter $\Gamma_{\text{min}}\approx 8$ μeV, which explains well the saturation observed below 40 K [Fig. 3(b)].

## V. CONCLUSION

We have presented a detailed study of the spin-wave dynamics in Cu_2_OSeO_3_ using small-angle neutron scattering, focusing on the temperature dependence of spin-wave stiffness and damping constants. Our findings demonstrate a significant enhancement in the sensitivity of SANS due to the larger sample size and the insulating nature of the material, enabling the detection of subtle features predicted theoretically but previously unresolved. The extracted exchange stiffness values show excellent agreement with established models, while the damping parameters highlight the low intrinsic spin-wave damping in Cu_2_OSeO_3_ compared to metallic chiral magnets. These results not only provide a comprehensive understanding of the helimagnon dynamics in Cu_2_OSeO_3_ but also establish the potential of advanced SANS techniques for probing spin-wave phenomena in other complex magnetic systems.

## Data Availability

The data that support the findings of this study are available from the corresponding author upon reasonable request and openly available in ILL data repository after the embargo period at https://doi.ill.fr/10.5291/ILL-DATA.5-41-1226 (Ref. 38).

## APPENDIX: ELASTIC SANS IN THE HELICAL STATE

Elastic SANS pattern measured at zero magnetic field at the base temperature T=2 K is shown in Fig. 4. The pattern manifests a two-domain helical state with the propagation vectors along [100] and [010] crystal axes, respectively.
